# Low-Dose Ionizing Radiation and Male Reproductive Immunity: Elucidating Subtle Modulations and Long-Term Health Implications

**DOI:** 10.3390/ijms26052269

**Published:** 2025-03-04

**Authors:** Jiacheng Yin, Yifan Ye, Yuankai Gao, Qing Xu, Muzhe Su, Shengkui Sun, Wenhui Xu, Qian Fu, An Wang, Sumin Hu

**Affiliations:** 1 School of Traditional Chinese Medicine, Beijing University of Chinese Medicine, Beijing 100029, China; 13294111895@163.com (J.Y.); yeyifan9411@163.com (Y.Y.); gykk20000817@163.com (Y.G.); xuqing0805@126.com (Q.X.); 20240931083@bucm.edu.cn (M.S.); sunshengkui123@163.com (S.S.); fuqian712@163.com (Q.F.); 2 Beijing Research Institute of Chinese Medicine, Beijing University of Chinese Medicine, Beijing 100029, China; xwh1428@126.com

**Keywords:** low-dose ionizing radiation, reproductive immunity, testicular immune microenvironment, epigenetic changes, male reproductive health

## Abstract

Low-dose ionizing radiation (LDIR) is a prevalent environmental factor with profound impacts on male reproductive health, particularly on the testicular immune microenvironment. This review examines the multifaceted effects of LDIR, emphasizing its ability to induce genotoxic stress, oxidative damage, and epigenetic modifications in reproductive cells. These alterations compromise DNA repair, disrupt chromatin structure, and induce immune dysregulation. Immune cells such as macrophages, T cells, natural killer cells, and dendritic cells exhibit significant functional changes under LDIR exposure, destabilizing the immune privilege critical for normal spermatogenesis. The long-term health implications of LDIR include impaired sperm quality, reduced fertility, and transgenerational risks through heritable genomic instability. This review underscores the importance of exploring the mechanisms underlying immune dysregulation and developing effective protective strategies. While LDIR’s full impact on male reproductive health remains to be elucidated, addressing the gaps in our understanding of immune microenvironmental changes is crucial for mitigating its adverse effects and improving reproductive health outcomes.

## 1. Introduction

Low-dose ionizing radiation (LDIR) is a ubiquitous environmental factor, originating from both natural sources, such as cosmic rays, terrestrial radiation, and radon gas, and anthropogenic sources, including medical imaging, radiation therapy, nuclear power generation, and occasional nuclear fallout [1,2]. Low doses are typically considered to be <100 mGy [3,4], although a fluctuating definition is found across the literature. Researchers have differing opinions on what constitutes a low dose, so this review does not provide a strict definition and instead determines the dose based on the descriptions provided by the authors of the original studies. While these exposures are generally considered low-risk compared to high-dose radiation, the cumulative and chronic nature of LDIR can pose significant health concerns, particularly for sensitive tissues like the male reproductive system. In this context, “long-term” refers to the genetic effects following radiation exposure.

Recent studies indicate that LDIR not only directly damages reproductive cells but also modulates the immune microenvironment of the male reproductive system, particularly the testis [5,6]. As an immune-privileged organ, the testis plays a vital role in balancing immune defense against infections and the maintenance of spermatogenesis. This delicate equilibrium is highly susceptible to disturbances from LDIR exposure, potentially leading to long-term reproductive health consequences. Understanding the biological effects of LDIR is essential for evaluating its long-term implications for male reproductive health. Current research suggests that ionizing radiation affects the testicular immune microenvironment through mechanisms such as DNA damage [3,7], epigenetic modifications, oxidative stress, and inflammatory responses [8]. These effects involve various immune cell types, including macrophages, T cells, dendritic cells, and natural killer cells [6,9,10]. However, the precise molecular pathways and cellular responses remain poorly understood, particularly in the context of low-dose radiation exposure [11]. Furthermore, there is limited understanding of the long-term and transgenerational effects of LDIR on the immune and reproductive systems, leaving critical knowledge gaps in the field [2,6,12].

This review aims to explore the specific impacts of LDIR on the immune microenvironment of the testis and examine the functional changes in immune cells induced by radiation. It discusses the response of immune cells to radiation and the broader implications for male reproductive health. Furthermore, it highlights the potential disruption of testicular immune privilege by LDIR and provides insights into mitigating its adverse effects on male fertility. See Abbreviations part for a full list of the abbreviations used.

## 2. Effects of Low-Dose Ionizing Radiation on Male Reproductive Health

LDIR can have adverse effects on male reproductive health, primarily through disruptions in testicular structure and sperm production. (More detailed information is summarized in the Supplementary Material Tables). Exposure to low doses of ionizing radiation results in reduced testis weight and sperm count, often by inducing DNA damage, apoptosis, and chromosomal aberrations in male germ cells. Additionally, LDIR leads to epigenetic modifications, including alterations in DNA methylation, histone modifications, and chromatin remodeling, which can persist across generations and exacerbate fertility issues. These epigenetic changes, along with direct DNA damage, contribute to long-term reproductive dysfunction, affecting both the individual and their offspring. The combined effects of these biological mechanisms highlight the complex and enduring nature of radiation-induced reproductive damage.

### 2.1. Spermatogenesis

Radiation-induced damage to the testicular tissue and germ cells primarily impairs spermatogenesis, resulting in reduced sperm count and quality, as well as an increased risk of infertility. Histological studies have demonstrated that LDIR reduces testis weight and sperm count, disrupts spermatogenesis, and induces degenerative changes in the seminiferous tubules [13]. These degenerative changes indicate a profound impairment of normal spermatogenic processes, as supported by recent findings [14]. Even at doses as low as 10 mGy, spermatogenesis can be arrested [13], while 75 mGy increases apoptosis in spermatogonial cells and spermatocytes [15]. Repeated single-dose exposure to 100 mGy leads to severe germ cell depletion, atrophy of seminiferous tubules, and a complete absence of mature sperm, indicating profound impairment of spermatogenic processes [13]. As the radiation dose increases, the severity and long-term consequences of testicular damage become more pronounced, with immune-mediated responses playing a significant role in later stages. After exposure to 30–50 cGy of radiation, lymphocytic infiltration in seminiferous tubules and interstitial tissues was observed after 5 years. Following exposure to 20–30 cGy of radiation, early autoimmune orchitis occurred, impairing spermatogenesis, with interstitial tissue infiltration detected 10–15 years later [16]. LDIR can impact DNA repair, genome integrity, and potentially promote tumor development [17,18]. A study comparing the gene networks in male and female germ cells under low-dose ionizing radiation found that the alterations in gene networks differed between sexes. Only myelocytomatosis oncogene (MYC) and cysteine-rich angiogenic protein 61 (CYR61) were shared in both male and female germ cells [6]. Thus, radiation-induced reproductive impairments are not only gender-specific but are also associated with distinct gene networks and pathways.

### 2.2. DNA Damage

Ionizing radiation causes significant DNA damage in male germ cells, leading to mutations, chromosomal aberrations, and impaired fertility. This damage can occur both directly through energy deposition in DNA molecules and indirectly via the generation of reactive oxygen species (ROS), which cause oxidative stress [7,19]. Spermatogonia, as the precursor cells for spermatogenesis, are particularly vulnerable to this radiation-induced DNA damage. LDIR has been shown to induce DNA double-strand breaks (DSBs) in spermatogonia, resulting in apoptosis, especially in undifferentiated spermatogonia. These cells undergo caspase-3-dependent apoptosis as a result of radiation exposure [20]. The key manifestations of radiation-induced genome instability (RIGI) in germ cells include DNA strand breaks, chromatin condensation, and chromosomal aberrations, all of which disrupt cellular functions and can trigger programmed cell death [21]. Given that spermatogonia are essential for the progression of spermatogenesis, their survival and efficient DNA repair are critical for maintaining fertility. However, DNA repair in spermatogonia is challenging due to their lack of sister chromatids. This leads to reliance on error-prone repair mechanisms such as non-homologous end joining (NHEJ), which increases the likelihood of mutations and genomic instability [22,23].

In addition to the direct DNA damage caused by radiation, the indirect effects of LDIR through ROS production further exacerbate cellular damage. ROS not only induce DNA damage but also damage lipids and proteins, amplifying the overall stress within the cell [7,19]. Although cellular repair mechanisms like homologous recombination and NHEJ are activated to address this damage, errors during repair can result in mutations and chromosomal instability, which are particularly harmful to germ cells. Since germ cells require genomic integrity for successful reproduction, these genotoxic effects can severely impair fertility. Furthermore, LDIR can alter the epigenetic landscape by modifying DNA methylation patterns, histone modifications, and chromatin structure. These alterations can disrupt the expression of critical genes involved in DNA repair, such as ATP-dependent helicase (ATRX) and MYC, potentially leading to heritable genomic instability and transgenerational effects [6].

### 2.3. Epigenetic Alterations and Long-Term Implications

The long-term consequences of LDIR include persistent declines in male fertility. Additionally, the genetic and epigenetic alterations in germ cells can be transmitted to offspring, raising concerns about transgenerational health risks. Addressing these challenges requires the identification of reliable biomarkers, such as ataxia telangiectasia mutated (ATM), checkpoint kinase 2 (CHK2), p53, and H2A histone family member X (H2AX) [24], for monitoring radiation exposure and understanding its effects. Future research must focus on the molecular pathways impacted by LDIR, particularly those involved in immune regulation, to develop effective protective strategies and mitigate its adverse effects on reproductive health.

The epigenetic alterations induced by LDIR, including changes in DNA methylation, histone modifications, and chromatin remodeling, play a critical role in long-term reproductive health outcomes [23,25,26,27]. Ye et al.’s study on human B lymphocytes found that after 4 weeks of low-dose radiation, B lymphoblastic HMy2.CIR cells exhibited global DNA hypermethylation [28]. These epigenetic alterations are of particular concern because they can persist across generations, affecting offspring without direct radiation exposure. Nakata et al.’s study demonstrated that testicular methylation dysregulation occurs following low-dose radiation exposure and suggested the possibility of certain genetic abnormalities [29]. The epigenetic changes brought about by LDIR can impair processes like fertilization and embryogenesis, with long-term consequences on reproductive health [30]. In the Daphnia magna model, low-dose ionizing radiation exposure induces transgenerational effects on DNA methylation. A small number of differentially methylated cytosines (DMCs) were found to persist across generations (e.g., two hypermethylated DMCs shared by F0, F2, and F3), highlighting the potential for germline-mediated epigenetic inheritance [31]. The genetic instability observed in the offspring of irradiated parents is associated with a comprehensive set of DNA damage present in their cells. In directly irradiated germ cells, alterations in DNA methylation patterns may generate strong epigenetic signals, which contribute to the transgenerational transmission of DNA damage to descendants. These radiation-induced signals in germ cells can influence offspring at the somatic level, manifesting as an increased frequency of DNA damage, likely mediated through inherited genetic or epigenetic changes [32]. Additionally, LDIR increases the risk of adverse birth effects, although these studies have focused on maternal exposure [2].

## 3. Effects of Low-Dose Ionizing Radiation on the Testicular Immune Microenvironment

**Figure 1:**
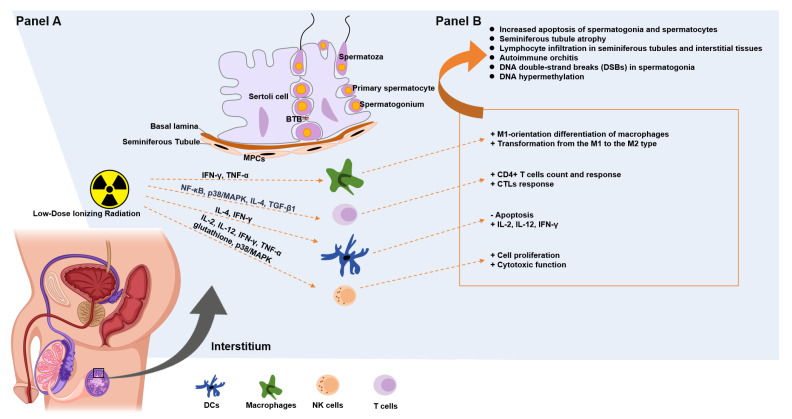
Effects of low-dose ionizing radiation on male testicular reproductive immunity. This figure summarizes the effects of low-dose ionizing radiation on the testicular immune microenvironment (**Panel B**) and the factors contributing to these effects (**Panel A**). (**Panel A**) Schematic representation of the impact of low-dose ionizing radiation on testicular immune cells and molecular signaling. Radiation exposure alters immune pathways, including pro-inflammatory and immunoregulatory cytokines [33,34,35] (e.g., IFN-γ, TNF-α, IL-4, IL-12, TGF-β), activation of signaling pathways [33,36,37] (e.g., NF-κB, p38/MAPK), and oxidative stress-related factors [33] (e.g., glutathione). These changes affect macrophages, T cells, and other immune components in the testicular interstitium. (**Panel B**) Summary of immune and pathological consequences induced by radiation exposure, including increased apoptosis of spermatogonia and spermatocytes, lymphocyte infiltration, autoimmune orchitis, DNA double-strand breaks, and DNA hypermethylation [13,15,16,20,28]. Additionally, specific immune alterations occur, such as macrophage M1 differentiation, shifts in CD4+ T cell and cytotoxic T lymphocyte (CTL) responses, and modulation of apoptosis [33,35,38,39,40,41,42,43,44,45,46,47]. BTB: Blood–Testis Barrier, TGF-β: Transforming Growth Factor Beta, IL-1β: Interleukin-1 Beta, FasL: Fas Ligand, LDIR: Low-Dose Ionizing Radiation, NK cells: Natural Killer Cells, DCs: Dendritic Cells, IFN-γ: Interferon-γ, TNF-α: Tumor Necrosis Factor-α, NF-κB: Nuclear Factor κB, p38/MAPK: P38-Mitogen-Activated Protein Kinases.

Since the testis is the primary site for the production of male germ cells, if testicular immune homeostasis is disrupted, it can lead to testicular inflammation and may even result in male infertility. This discussion mainly outlines the effects of LDIR on the testicular immune response. The pathways affected are summarized in Figure 1, while Supplementary Tables S1 and S2 consolidate these effects. There are various types of immune cells within the testis, and LDIR affects these cells, thereby influencing reproductive immunity. Macrophages, dendritic cells, and T cells are all immune cells that can impact testicular function. Sertoli cells express several immunoregulatory factors and can influence the actions and functions of these different immune cells to maintain protection for immunogenic germ cells.

### 3.1. Low-Dose Ionizing Radiation-Induced Changes in Immune Cell Populations

#### 3.1.1. Natural Killer Cells

In male reproductive immunity, NK cells are essential for immune surveillance against infections caused by viruses, bacteria, fungi, and protozoa. Within the male reproductive system, particularly in the testes, NK cells contribute to protecting the tissue from infections that could impair spermatogenesis and fertility [48]. However, their activity must be tightly regulated to avoid damaging the immunologically privileged environment of the testes. The NK cells in the male reproductive tract secrete pro-inflammatory cytokines and exhibit cytotoxic activity to eliminate infected or transformed cells, helping to control infections. However, when NK cell activity becomes excessive, it can jeopardize the immune privilege necessary for tolerance towards sperm cells. This imbalance may result in testicular autoimmune diseases such as orchitis, which can adversely impact spermatogenesis and fertility [49]. Studies have shown that LDIR can enhance NK cell activity by stimulating cell proliferation and promoting the cytotoxic functions of NK cells [33,38]. This enhanced activity might influence the immune environment in the testes, with potential implications for both fertility and the control of testicular tumors. LDIR can also affect NK cell-mediated cytotoxicity indirectly by activating the endocrine and central nervous systems [39]. Given the close interplay between the endocrine system and reproductive function, this influence could affect testicular immune responses and potentially impact fertility. Although many studies have reported LDIR-induced NK cell activation, the molecular mechanisms underlying this process remain unclear and controversial. Understanding these mechanisms in the context of male reproductive health is crucial for evaluating how LDIR might impact both fertility and testicular immune defense. The possible mechanisms for LDIR-induced NK cell activity enhancement are related to the increased production of glutathione and the elevated secretion of cytokines such as IL-2, IL-12, interferon-γ (IFN-γ), and tumor necrosis factor-α (TNF-α) [34]. In the testicular environment, these cytokines can modulate immune responses in a way that balances protection against infections with the preservation of spermatozoa from immune attack. Glutathione is upregulated by cells to counteract oxidative stress, primarily due to LDIR (low-dose ionizing radiation)-induced ROS, including superoxide anions (O_2_⁻), hydrogen peroxide (H_2_O_2_), and hydroxyl radicals (·OH), which trigger intracellular oxidative stress [50]. ROS play a regulatory role in the signaling pathways within NK cells, acting as signaling molecules that initiate critical pathways, such as phosphatidylinositol 3-kinase/AKT serine/threonine kinase (PI3K/AKT) and mitogen-activated protein kinase (MAPK), to enhance NK cell functionality [51,52]. This regulation contributes to improved metabolic adaptation and effector functions, including cytotoxicity and cytokine production. At moderate levels, ROS can promote these beneficial effects by activating these pathways, while glutathione plays a complementary role in maintaining ROS at optimal levels that support signaling without causing oxidative damage. However, excessive ROS may overwhelm the antioxidative capacity of cells, leading to oxidative stress that disrupts mitochondrial function, reduces adenosine triphosphate (ATP) availability, and ultimately impairs the cytotoxic and immune-regulatory capabilities of NK cells [53]. These insights underscore the importance of a balanced ROS level for the optimal regulation of NK cell activity under LDIR conditions. Research by others found that LDIR-induced NK cell activation is associated with the P38-mitogen-activated protein kinases (p38/MAPK) (mitogen-activated protein kinase) signaling pathway [33]. This pathway’s role in testicular immune regulation could be significant, influencing both immune surveillance and the protection of reproductive function. Further research is needed to elucidate the exact mechanisms of LDIR-induced NK cell activation to apply this effect to the treatment of immune-related diseases.

#### 3.1.2. Macrophages

Macrophages play a crucial role in the clearance of pathogens and the maintenance of tissue homeostasis. In the context of male reproductive immunity, testicular macrophages are pivotal in preserving the immunosuppressive environment required for normal spermatogenesis, while also protecting against infections that could impair fertility [54]. Specific tissue and microenvironmental signals trigger macrophage differentiation into two subpopulations: immunostimulatory macrophages (or M1 macrophages, M1) and immunoregulatory macrophages (or M2 macrophages, M2). A balance between M1 and M2 macrophages is crucial to maintaining immune privilege and supporting tissue integrity without initiating detrimental inflammatory responses [55]. M1 macrophages can activate type 1 helper T cells (Th1) to enhance the immune response [56], which, in the testes, could potentially disrupt the delicate environment needed for sperm production if not tightly regulated. M2 macrophages mediate anti-inflammatory responses through type 2 helper T cells (Th2) and promote tissue remodeling processes such as angiogenesis [57]. Additionally, M2 macrophages promote tumor cell growth, angiogenesis, invasion, and metastasis [58,59] and are referred to as tumor-associated macrophages (TAMs). In testicular cancer, TAMs can play a role in tumor progression, making the regulation of macrophage polarization a potential therapeutic target [60,61]. Reports indicate that LDIR affects the transformation of different macrophage types. LDIR might influence the balance of macrophage populations within the testes, potentially affecting both fertility and susceptibility to testicular tumors. Nadella and Prakash et al. found that LDIR induces M1-associated effector cytokines and reduces pro-tumor and M2-associated effector cytokines [40,41], thereby promoting the differentiation of TAMs into the M1 phenotype. LDIR programmably induces the differentiation of inducible nitric oxide synthase (iNOS) M1 macrophages, which coordinate the recruitment of cytotoxic T cells into solid tumors and their killing within the tumors. This shift could have implications for the immune response within the testes, potentially influencing both inflammation and immune tolerance mechanisms. Simultaneously, LDIR can induce the transition of M1 phenotypes to M2 macrophages [42]. In the testes, this transition might contribute to a protective anti-inflammatory state but also raises concerns about the potential for impaired immune surveillance against infections or malignancies. The mechanism of LDIR-induced macrophage differentiation is related to the induction of endothelial activation and the expression of Th1 chemokines, as well as the inhibition of angiogenic factors, immunosuppressive factors, and tumor growth factors. These mechanisms could similarly affect the testicular microenvironment, with potential consequences for both immune protection and fertility. Thus, the pathways through which LDIR affects macrophage function also include the inhibition of the iNOS pathway and nitric oxide (NO) production, reduction of oxidative burst activity and superoxide production, and inhibition of protein kinase-B (AKT) and p38/MAPK phosphorylation [62,63,64]. These pathways are likely significant in the testicular environment, where oxidative stress and inflammation need to be carefully regulated to avoid damaging the delicate process of spermatogenesis. These in vivo and in vitro study results provide strong evidence for the effects of LDIR on macrophage differentiation and function. In the context of male reproductive immunity, understanding these effects is crucial for developing strategies to protect fertility while managing testicular inflammation.

#### 3.1.3. Dendritic Cells

Dendritic cells (DCs) are the most efficient and specialized antigen-presenting cells (APCs) in the innate immune system and are capable of initiating adaptive immune responses [65]. DCs help maintain immune tolerance and prevent autoimmune attacks on developing sperm [66,67]. In tumor-bearing mice, low-dose ionizing radiation can moderately reduce the apoptosis of dendritic cells; this effect has been found to be associated with alterations in the cytokine milieu, including partial downregulation of IL-4 and IFN-γ [35]. Persa et al. found that irradiation increased the production of IL-1α and IL-1β by dendritic cells in vivo [68]. Shigematsu et al. reported that pre-irradiating DCs with 0.05 Gy of LDIR resulted in the highest T cell proliferation capacity and increased the production of IL-2, IL-12, and IFN-γ. Increased IL-12 and IFN-γ levels can be particularly relevant in the testicular environment, where inflammatory cytokines may disrupt the immune privilege status of the testes, leading to impaired sperm production. However, LDIR did not enhance the expression of major histocompatibility complexes (MHCs) or co-stimulatory molecules on DCs, such as cluster of differentiation 1a (CD1a), CD40, CD80, CD86, and intracellular adhesion molecule (ICAM) [43]. This suggests that reports on the effects of LDIR on DCs are contradictory, and the mechanisms behind these results remain to be elucidated. What is certain is that LDIR can stimulate innate immune cells, thereby further activating adaptive immune cells to enhance immune responses. In the male reproductive system, this could theoretically lead to an imbalance in immune privilege, thus influencing fertility outcomes. Notably, inter-laboratory differences in irradiation dose, dose rate, and irradiation time may account for these conflicting results, indicating a need for further research to determine the effects and mechanisms of LDIR on DCs. In particular, the role of LDIR in modulating immune responses in immune-privileged organs like the testes remains an important area of investigation to understand its potential impact on male fertility.

#### 3.1.4. T Cells

T cells are at the core of cell-mediated immunity. LDIR can increase the subsets of CD4+ T cells and enhance their response [44,45], and it also enhances the response of CD8+ cytotoxic T lymphocytes (CTLs) [46,47]. T cell subsets, especially CD4+ T helper cells, play a crucial role in modulating responses to sperm antigens, potentially contributing to conditions like autoimmune orchitis or the generation of anti-sperm antibodies (ASA). The activation of survival/signaling proteins such as nuclear factor κB (NF-κB) and p38/MAPK, along with the increased ability of T cells to produce immune-enhancing cytokines such as IL-4 and the reduced production of major immunosuppressive cytokines (transforming growth factor β1, TGF-β1), are observed changes that may represent the molecular mechanisms by which LDIR modulates T cell immunity [36,37]. Studies have shown that the NF-κB and MAPK pathways are crucial in regulating inflammation and immune responses within the testes. NF-κB, in particular, has been implicated in modulating local inflammatory responses to prevent autoimmune attacks against sperm cells, while p38/MAPK is associated with promoting T cell survival and function under stress conditions, such as those induced by oxidative damage or infections in the male reproductive tract [69,70]. This signaling cascade is relevant in male reproductive health, where a delicate balance between pro-inflammatory and anti-inflammatory cytokines influences testicular immune privilege and sperm tolerance. It has been reported that these signaling network changes enhance T cell immunity by increasing the expression levels of several CD markers and chemokines, such as T cell receptor (TCR), CD2, CD3, CD4, and CD28 [71,72,73]. Research indicates that increased expression of CD3 and CD28 is critical for the activation of T cells during immune responses [74,75]. In the male reproductive system, inappropriate T cell activation through these markers has been associated with autoimmune orchitis and the generation of ASA, which can impair fertility. Additionally, chemokines like CXC chemokine ligand 10 (CXCL10) and CXC chemokine ligand 12 (CXCL12), often upregulated during inflammation, have been shown to attract T cells to sites of inflammation, including the testes, further exacerbating autoimmune conditions in reproductive tissues [74]. The expression of these markers is significant in reproductive immunology, as upregulation of TCR-associated molecules could enhance the T cell recognition of sperm antigens, potentially leading to autoimmune responses in the male reproductive system. Wang et al. found a moderate decrease in the cell surface expression of cytotoxic T lymphocyte-associated antigen-4 (CTLA-4) on mouse Tregs [76]. The mechanisms underlying LDIR-induced Treg effects have not been well explored; these phenomena may be part of the mechanisms by which LDIR enhances Treg immune responses. LDIR can stimulate the selective retention or expansion of Tregs capable of producing immunosuppressive activity to control autoimmune diseases, as observed in animal models of autoimmune diseases [77]. This immunosuppressive activity of Tregs highlights the complex role of LDIR in both reproductive health and autoimmune regulation. In male reproductive immunity, the effect of LDIR on Tregs could vary depending on the specific immune context within the testes, potentially influencing outcomes like fertility or the development of reproductive autoimmune disorders.

### 3.2. Low-Dose Ionizing Radiation-Induced Changes in Cytokine

IL-10 plays a critical role in maintaining immune privilege in the testes by suppressing local immune responses that could otherwise target sperm as foreign antigens. Studies have shown that reduced levels of IL-10 are associated with increased testicular inflammation and disruption of the blood–testis barrier, a structure crucial for preventing immune cells from accessing sperm. In animal models, decreased IL-10 expression has been linked to the development of autoimmune orchitis and increased production of pro-inflammatory cytokines, which can lead to infertility [78]. Long-term exposure to LDIR in miners has been found to result in an increased expression of IL-10 [79]. Hussien et al. found that serum IL-10 expression increased in the group that received low initial doses of irradiation compared to the group that did not receive such doses [63]. Increased IL-10 levels could potentially protect sperm from immune-mediated damage, suggesting that LDIR might modulate reproductive immunity in a dose-dependent manner.

In addition, LDIR has been shown to induce an increase in IL-2 levels [34]. LDIR promotes the production of IL-2 in dendritic cells [43]. One study suggests that LDIR can enhance immune function in NK cells, and it was found that pretreatment with low-dose IL-2 prior to low-dose radiation exposure enhances the cytotoxic activity of NK cells [80]. Low-dose radiation (75–150 mGy) significantly affects the expansion and secretion of effector proteins (such as IFN-γ and TNF-α) in NK cells through the p38/MAPK pathway. Pre-radiation treatment with low-dose IL-2 can further enhance this effect [33,80]. The findings revealed that radiation exposure led to an increased production of IL-12 by dendritic cells (DCs). Further mechanistic analysis indicated that the upregulation of IL-12 was mediated through the activation of the ATM/NF-κB signaling pathway [81]. Additionally, LDIR can induce adaptive responses in the body, enabling it to partially mitigate the effects of subsequent high-dose radiation exposure. In studies on splenic lymphocytes in mice, it was found that when low-dose radiation was used as a priming dose prior to high-dose radiation, it significantly attenuated the elevation of IL-2 levels and enhanced immunosuppression. The mitogen-activated protein kinase/extracellular regulatory kinase (MAPK/ERK) and stress-activated protein kinase/c-Jun NH2-terminal kinase (SAPK/JNK) signaling pathways were found to play crucial roles in the activation of T lymphocytes and the promotion of IL-2 production [82]. Similarly, studies in rats have demonstrated that exposure to 250 mGy of ionizing radiation could mitigate the elevation of serum IL-2 levels induced by 500 mGy of ionizing radiation [83].

LDIR also plays a crucial role in the regulation of other cytokines. IL-6 interacts with steroidogenesis and regulates the production of hormones by the adrenal glands and testes that are essential for survival. Under pathological conditions, such as autoimmune diseases, high levels of IL-6 are found in semen, where these cytokines play a key role in immune regulation within the male gonads [84,85]. TNF-α can induce apoptosis, inhibit spermatogenesis, and disrupt steroid hormone synthesis. High concentrations of TNF-α may lead to chronic testicular damage, which in turn affects both sperm count and quality [86]. TGF-β helps maintain the integrity of the blood–testis barrier and is crucial for the process of sperm maturation. Studies have also indicated that blocking TGF-β signaling could be an effective strategy for treating certain types of testicular infections [87]. IFN-γ can activate macrophages, enhance antigen presentation, and upregulate MHC molecule expression. However, excessive expression of IFN-γ within the testes may trigger inflammation and autoimmune diseases [88]. IFN-γ, IL-10, IL-6, and TNF-α coordinate the inflammatory response. Long-term exposure to low-dose ionizing radiation in miners has been shown to increase the expression of IFN-γ, IL-10, IL-6, and TNF-α [79], providing supporting evidence for the persistent inflammatory response induced by prolonged low-dose ionizing radiation exposure [89]. IL-1β is a key pro-inflammatory cytokine that can induce apoptosis, inhibit spermatogenesis, and disrupt the synthesis of steroid hormones. Studies have shown that activated macrophages exposed to 100 mGy of ionizing radiation exhibit decreased IL-1β secretion [90]. Additionally, in the evaluation of ionizing radiation’s effects on atherosclerosis, it was found that in response to 50 mGy exposure, bone marrow-derived M0 and M2 macrophages also showed reduced IL-1β secretion [91]. Moreover, in hepatocytes, while LDIR does not directly alter IL-1β levels, it can mitigate the increase in IL-1β levels induced by high glucose (HG) [92]. This suggests that short-term low-dose ionizing radiation can downregulate inflammation. Therefore, short-term low-dose ionizing radiation exposure may be used clinically to treat benign inflammatory diseases [93,94].

To better illustrate the biological effects of different radiation doses, we have summarized the effects of ≤100 mGy and >100 mGy in Table 1. Studies have shown that radiation exposure at 7.97 mGy is associated with an increase in LINE-1 methylation levels, whereas at 157.74 mGy, global DNA methylation levels tend to decrease [27]. These alterations may influence gene expression and genomic stability. Additionally, radiation affects macrophage activation, as evidenced by increased TNF-α secretion at 100 mGy or 200 mGy [39], while at 250 mGy, TNF-α levels are reduced [63]. These findings suggest that different radiation doses can induce distinct biological responses that may have implications for immune regulation and epigenetic modifications. Further research is needed to explore the mechanisms underlying these dose-dependent effects.

## 4. Conclusions

LDIR can significantly alter the immune landscape of the testes, with potential repercussions for male reproductive health. These alterations include increased DNA damage, epigenetic changes, and disruptions to the immune balance necessary for normal spermatogenesis. The blood–testis barrier and testicular immune privilege are particularly vulnerable to LDIR, which can lead to impaired fertility and increased risk of autoimmune responses against sperm cells.

The evidence points to a nuanced relationship between LDIR and male reproductive immunity, where even low levels of radiation can trigger significant biological responses. These responses, which are associated with various immune cells such as NK cells, macrophages, dendritic cells, and T cells, play distinct roles in maintaining testicular health and responding to radiation-induced stress and are closely linked to the regulation of cytokines that modulate inflammation and immune tolerance within the testes. Several critical questions remain unanswered, including the precise molecular mechanisms by which LDIR influences immune cell function and how these changes translate into clinical and sub-clinical outcomes. Understanding the sub-clinical changes may be crucial for identifying the early biomarkers of radiation exposure and long-term health implications. Future research will require a more refined and intelligent dose concept considering the distinct biological effects that may arise at different dose ranges. Additionally, the long-term and transgenerational effects of LDIR on male reproductive health warrant further investigation. Future research should focus on elucidating these mechanisms and developing strategies to protect and mitigate the adverse effects of LDIR on male reproductive health.

In conclusion, this review underscores the importance of understanding the subtle yet profound impacts of LDIR on male reproductive immunity. Given the increasing exposure to low levels of radiation in modern life, it is imperative to deepen our understanding of these effects and to develop effective interventions to safeguard reproductive health across generations.

## Figures and Tables

**Table 1:** Comparison of the biological effects of ≤100 mGy and >100 mGy radiation doses.

	≤100 mGy	>100 mGy
Methylation Changes	Methylation levels of LINE-1 increased. (7.97 mGy [27])	Global DNA methylation levels decreased. (157.74 mGy [27])
Macrophages Activation	Increased TNF-α secretion. (100 mGy or 200 mGy [39])	Decreased TNF-α levels. (250 mGy [63])

## Supplementary Materials

The following supporting information can be downloaded at: https://www.mdpi.com/article/10.3390/ijms26052269/s1.

## Abbreviations

The following abbreviations are used in this manuscript:

Abbreviation	Full Name
AKT	protein kinase-B
APCs	antigen-presenting cells
ASA	anti-sperm antibodies
ATM	ataxia telangiectasia mutated
ATP	adenosine triphosphate
ATRX	ATP-dependent helicase
BTB	blood–testis barrier
CD1a	cluster of differentiation 1a
CHK2	checkpoint kinase 2
CTLA-4	cytotoxic T lymphocyte-associated antigen-4
CTLs	cytotoxic T lymphocytes
CXCL10	CXC chemokine ligands 10
CXCL12	CXC chemokine ligands 12
CYR61	cysteine-rich angiogenic protein 61
DCs	dendritic cells
DMCs	differentially methylated cytosines
DSBs	double-strand breaks
EAO	experimental autoimmune orchitis
Fas/FasL	Fas/Fas ligand
Gas6/ProS-TAM	growth arrest-specific gene 6/protein S-Tyro3, Axl, and Mer
H2AX	H2A histone family member X
H_2_O_2_	hydrogen peroxide
HG	high glucose
ICAM	intracellular adhesion molecule
IFN-γ	interferon-γ
iNOS	nitric oxide synthase
LDIR	low-dose ionizing radiation
MAPK	mitogen-activated protein kinase
MAPK/ERK	mitogen-activated protein kinase/extracellular regulatory kinase
MHCs	major histocompatibility complexes
MYC	myelocytomatosis oncogene
NF-κB	nuclear factor κB
NHEJ	non-homologous end joining
NO	nitric oxide
O_2_⁻	superoxide anions
LINE-1	long interspersed nuclear element-1
·OH	hydroxyl radicals
p38/MAPK	P38-mitogen-activated protein kinases
PD-1/PD-L1	programmed death-1/programmed death-ligand 1
PI3K/AKT	phosphatidylinositol 3-kinase/AKT serine/threonine kinase
PRRs	pattern recognition receptors
RIGI	radiation-induced genome instability
ROS	reactive oxygen specie
SAPK/JNK	stress-activated protein kinase/c-Jun NH2-terminal kinase
SCs	sertoli cells
TAMs	tumor-associated macrophages
TCR	T cell receptor
TGF	transforming growth factors
Th1	type 1 helper T cells
Th2	type 2 helper T cells
TNF-α	tumor necrosis factor-α
Tregs	regulatory T cells
